# Correction to: Steroid receptor coactivator‐1 enhances the stemness of glioblastoma by activating long noncoding RNA XIST/miR‐152/KLF4 pathway

**DOI:** 10.1111/cas.16026

**Published:** 2023-12-13

[Miaomiao Gong, Xun Wang, Lin Mu, Yueyue Wang, Jinjin Pan, Xiaocheng Yuan, Haoran Zhou, Jinshan Xing, Rui Wang, Jian Sun, Qiwang Liu, Xiya Zhang, Lin Wang, Yiying Chen, Yandong Pei, Shao Li, Liang Liu, Yongshun Zhao, Yuhui Yuan. Steroid receptor coactivator‐1 enhances the stemness of glioblastoma by activating long noncoding RNA XIST/miR‐152/KLF4 pathway. *Cancer Sci*. 2021; 112:604–618. https://doi.org/10.1111/cas.14685.]

The photos in Figure 4(D) include an error. Some of the areas in Figure 4(A) and 4(D) of this paper are similar. The corrected Figure 4(D) is shown below:

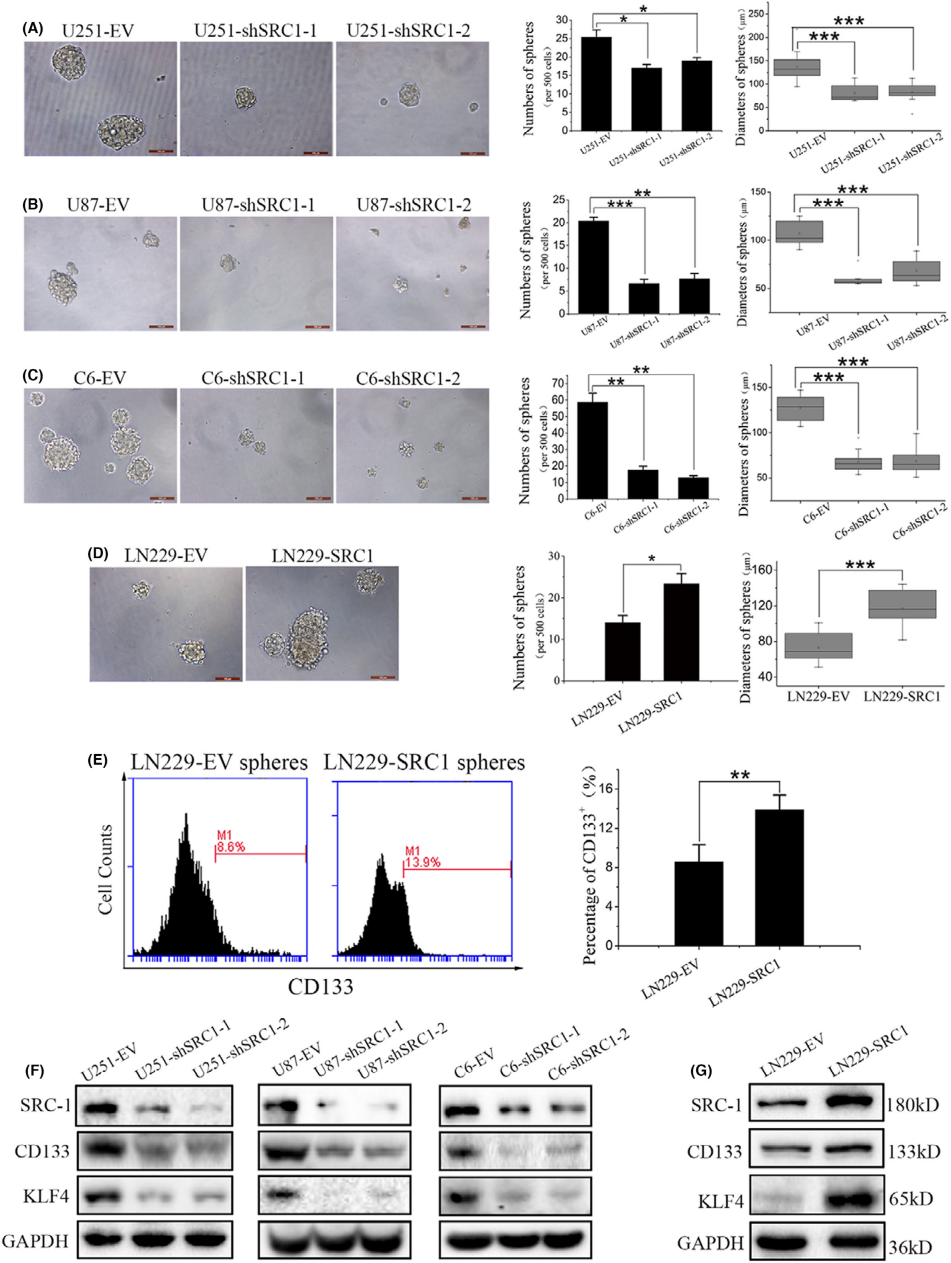

We apologize for this error.

